# In medio stat virtus? A reply to Dupras

**DOI:** 10.1186/s43682-023-00017-1

**Published:** 2023-05-18

**Authors:** Luca Chiapperino, Francesco Paneni

**Affiliations:** 1 STS Lab, Institute of Social Sciences, Faculty of Social and Political Sciences, University of Lausanne, Quartier UNIL-Mouline, Bâtiment Géopolis, Bureau 5556, Lausanne, CH-1015 Switzerland; 2 Center for Translational and Experimental Cardiology (CTEC), University Hospital Zurich and University of Zurich, Zurich, CH-8091 Switzerland; 3 Department of Cardiology, University Heart Center, University Hospital Zurich, Zürich, Switzerland

We are grateful towards Charles Dupras for having provided a critical assessment of our paper *Why epigenetics is (not) a biosocial science and why that matters*. We wish to reciprocate sympathy towards his well-structured and articulated proposal to reconsider widespread assumptions about the distinctions between epigenetics and genetics. As we pointed out in our reply to Silvio Zaina’s commentary, the biological relationship between genetics and epigenetics requires a more nuanced treatment than the one we could offer in our paper. Nonetheless, we wish to clarify that assimilating the former to the factors of nature and the latter to the factors of nurture was not part of our argument towards a better uptake of the biosocial dimensions of epigenetic research. Neither was it to cut a clear distinction between “biosocial” as a qualifier of epigenetics as necessarily opposed to genetics. The developmental version of epigenetics we argue for—as do others [1, 2]—pleads towards moving beyond such dichotomies. The core of our proposal has in fact been largely explored at a conceptual level [3, 4]: the problem is not whether (genetic or epigenetic) processes of development, health differentiation, and evolution are *either* nature *or* nurture, whether they are *either* biosocial *or* not. We are asking a rather different core question: To what extent do the methods, tools, and study designs of epigenetics capture the interactions that necessarily occur between nature and nurture, or even between biological and social processes? In answering this question, one should not suppose wrongly that we hold another problematic dichotomy.

Dupras’ reading of our paper is that of a call for “less reductionism and more holistic methods” in epigenetics research [5]. Here, again, nuance is paramount.^1^ On p.3, we underline that the full complexity of biosocial processes may be incommensurable to the methods of biomedical sciences. Yet, on p.4, we explicitly affirm that our paper is “also not a call for a foundational endeavor leading to an alleged holistic biosocial science” capable of seizing this complexity in full. We position ourselves in between the rejection of reductionist methods to apprehend biosocial health differentiation and the foundational endeavor of a holistic biosocial science. As the title of this reply suggests, *in medio stat virtus.* Our position builds on distinct threads of research in the history, philosophy, and socio-anthropology of science that consider the dichotomy reductionism/holism inadequate to understand postgenomic scientific practices and their relation to biosocial processes of health differentiation [6–8]. The point is not whether these methods are reductionist or not (they are), but how they “operate most prolifically at the fuzzy boundary between the trivial and the complex” [6]. To make the point in a more concrete way, one could conceive of a proliferation of reductionist methods that go in the direction of addressing (notice, *addressing* not seizing holistically) the complexity of the epigenome we point to. Take, as an example, measurements of stress or allostatic load that have been widely employed in epigenetic studies of the effects of psychosocial environments on health and disease [9, 10]. Most of the critique of these methods has focused on their lacking definition, standardization, and fidelity [11, 12] or on the molecularization of biography and milieu [13] they operate. These methods are certainly reductionist, but are they also doomed to provide a poor picture of the environmental embeddedness of our health? Is a different imbrication of the biological, psychological, and social determinants of health possible starting from them? Our paper points out that a positive answer to this question is possible. These methods are in fact embedded into study designs that reproduce a simplistic linearity of biosocial processes, often going as follows: exposures (which can be objectivised with such measurements of stress and/or allostatic load) produce/activate biological differences (readable in the epigenome), which are in turn linked (mechanistically and/or through statistical associations) to disease. Yet, one could easily imagine a different configuration employing these same methods, which would not suffer from the same problem of simplistic linearity. For instance, repeated measures of such psychological and/or physiological scales could provide evidence of (again, *not seize holistically*) the multi-directional and longitudinal effects of stress, its biological embodiment, its biopsychosocial looping effects, etc. This different configuration would leave untouched the reductionist qualities of the methods of epigenetics (i.e., measurements of stress or allostatic load, much like molecular techniques), but would complexify the account of the phenomenon these methods and tools can offer. Our point has never been to indicate a unique path, or to validate, define, or certify whether epigenetics is a holistic biosocial science. Nor it has been to “implicitly condemn genetics to the realm of “non-biosocial” sciences” [5]. Rather, our concern lies in documenting *how* the methods, tools, and study designs of the field—where committed to such questions—could possibly explore the mixed biological and socio-environmental determinants of epigenetic differences.

The objective of our paper was also *not* to criticize those “ambitious” initiatives cited by Dupras, such as the International Human Epigenome Consortium (IHEC) [5]. Nor to take any stance on what sub-field of epigenetics, as cited by Dupras, matters the most. We do not question the merit and importance of IHEC for the advancement of standards, methods, reference knowledge, or even the sense of community for people working in epigenetics on a global scale. Neither do we deny that the boundary between epigenetics and genetics is “blurred” [5]. However, while theoretically minor and frugal, the point we raise can be relevant for endeavors aiming to provide reference epigenomes for the understanding of health and disease. Definitional controversies notwithstanding,^2^ few hold the position that interindividual differences in the epigenome (and their health consequences) can be fully recapitulated by genetic variation, including the variation across so-called genetic ancestry groups. It is likely, as Dupras himself argues, that *some* traits are very stable over time, while some *others* are relatively plastic; *some* may “even be considered innate rather than acquired,” [5] while some *others* may be heavily affected by environmental or social exposures. Let us stick to ethnicity to make a concrete example: the evidence is inconclusive on a trait-by-trait basis, but some have shown that, across the genome, genetic ancestry can account for about three-quarters of the association between ethnicity and methylation differences [15]. The remaining quarter being attributed to what the study authors call shared environmental, social, and cultural factors within a given group. There is, in this bit of data, the suggestion that *both* differences in genetic ancestry *and* the social construct of cultural and ethnic differences could explain epigenetically measurable disparities in disease prevalence and health trajectories across different groups of people. This means that ongoing calls towards the promotion of diversity and inclusion of wider reference genetic populations in reference epigenomic maps and epigenome-wide association studies [16, 17] are indeed much-needed. This evidence certainly calls for expanding epigenomic databases beyond the current prevalence of data produced from participants of European ancestry. Yet, we ask: what is currently on the agenda of the global epigenetics community to account for that share of health disparities across social groups that are due to differences in relevant social and environmental exposures? It is likely that the biosocial dimensions of ethnic differences in the epigenome do not have a fixed, either biological or social ontology [cf. 18]. Perhaps, producing the reference epigenomes of certain traits will be highly sensitive to genetic differences across and within ancestry groups. Perhaps for other traits, reference epigenomes will be more open to varied developmental trajectories, local contexts, or even culturally situated practices. Of note, one could argue with Meloni and colleagues that even simplistic cause-effect models may be a problematic form of biosocial determinism around these epigenetic differences [19]. The point we raise is a call to the global epigenetic community to refrain from overlooking either side of this biosocial continuum: its multiple causal pathways and the mixed processes of biological and social differentiation that manifest in the epigenome. One does not need to embrace an essentialist social *or* biological view of ethnic differences in the epigenome (to stick to the example chosen here): our point is, to reiterate, one of balance and symmetry in the development of tools, methods, and study designs of epigenetic research. There might be more than the imputation of epigenetic differences from genetic variation to the improvement of diversity and inclusion in epigenetic study designs. In this call to symmetry lies—ambiguities and limitations notwithstanding—another concrete way to operationalize our proposal. The simplistic stance may be of those who need to pick a side on what is, in the end, a fabricated dichotomy between the biological or social origins of epigenetic differences. Again, *in medio stat virtus*.

## Data Availability

Data sharing is not applicable to this article as no datasets were exclusively generated for the current study.

## Footnotes

1 We are working under the assumption that Dupras’ reference to the reductionism vs. holism debate is here meant to underline an explanatory distinction, although the author does not clarify it. We take a reductionist position to hold, in Dupras' view, that facts about the mixed biological and social origins of disease can (at least in part, if not fully) be explained by the methods of epigenetics. And, conversely, an allegedly holistic position (which he attributes to us) to claim instead that explanations of higher-level facts, such as the biosocial determinants of health, cannot benefit from the current reductionist methods, tools, and study designs of epigenetics.

2 For one of the latest takes on these definitional issues, see the recent article by Bernhard Horsthemke on *Clinical Epigenetics* [14].
